# Synergistic effect of *Bacillus subtilis* and *Paecilomyces lilacinus* in alleviating soil degradation and improving watermelon yield

**DOI:** 10.3389/fmicb.2022.1101975

**Published:** 2023-01-13

**Authors:** Peng Chen, Jinglei Zhang, Mei Li, Feng Fang, Jindong Hu, Zuowen Sun, Ansheng Zhang, Xingxiang Gao, Jian Li

**Affiliations:** ^1^Institute of Plant Protection, Shandong Academy of Agricultural Sciences, Jinan, China; ^2^Key Laboratory of Natural Enemies Insects, Ministry of Agriculture and Rural Affairs, Jinan, China; ^3^Shandong Provincial Engineering Technology Research Center on Biocontrol of Crop Diseases and Insect Pest, Jinan, China; ^4^Shandong University of Traditional Chinese Medicine, Jinan, China; ^5^Shandong Provincial Key Laboratory of Applied Microbiology, Ecology Institute, Qilu University of Technology (Shandong Academy of Sciences), Jinan, China; ^6^Department of Plant Protection, Shandong Agricultural Technology Extension Center, Jinan, China

**Keywords:** continuous cropping, microbial biofertilizers, soil quality, microbial community, watermelon

## Abstract

Continuous cropping of watermelon (*Citrullus lanatus*) may lead to soil degradation. As a soil conditioner, microbial agent has great potential in improving soil function and enhancing plant growth. In this study, we aimed to explore how microbial agent relieves the soil sickness of watermelon by analyzing watermelon performance, soil physicochemical properties and microbial community structures. Results suggested that microbial agent treatments significantly changed the photosynthetic efficiency of upper and lower leaves, which helped improve the growth of watermelon. The single fruit weight, fruit sugar degree and total phosphorus of soil following treatment with a mixture of *Paecilomyces lilacinus* DZ910 and *Bacillus subtilis* KC1723 (treatment D_K) were higher than those in single biofertilizer treatments and control. The soil microbial community under microbial agent treatments also changed significantly, indicating the feasibility of using microbial agents as soil remediations. The proportions of *Pseudomonas* and *Flavobacterium*, changed significantly after using microbial agents. *Pseudomonas* increased significantly after *B. subtilis* KC1723 and D_K treatments, while *Flavobacterium* increased significantly after using all three kinds of microbial agents compared to control. Increases in these bacteria were positively correlated with agronomic variables of watermelon. The fungi *Aspergillus* and *Neocosmospora* in the soil, which create an soil sickness of watermelon, decreased after KC1723 and D_K treatments. Meanwhile, *Aspergillus* and *Neocosmospora* were positively related to *Myceliophthora* incidence and negatively correlated with watermelon growth (single fruit weight and photosynthetic efficiency of upper leaves). Our microbial agent, especially D_K, represents a useful technique for alleviating soil sickness in watermelon.

## Introduction

Watermelon (*Citrullus lanatus*) is an annual plant belonging to the family of *Cucurbitaceae* and is an important fruit crop grown worldwide (Guo et al., 2013). Watermelon flesh is rich in nutritional compounds, such as sugars, vitamins and other minerals that play important roles in human metabolism (Ding et al., 2021). Because of the high economic benefit, watermelon has been cultivated continuously in the same fields by farmers. Previous studies have shown that long-term continuous cropping may cause changes in soil fertility, enzyme activities, microbial communities and nematode communities (Chen et al., 2020; Zla et al., 2020).

Microbial agents, such as biopesticides and biofertilizers, are becoming widely used by people in agricultural practice, and should be expected to play an increasing role in the development of sustainable agriculture (Kowalska et al., 2020). Microorganisms are material and energy carriers in the soil and also essential indicators of soil health (Hermans et al., 2017; Mcik et al., 2020; Chen et al., 2022). Healthy growth of crops is inseparable from a healthy soil microbial community structure (Chen et al., 2022). Soil microbial community structure in agricultural production is mainly influenced by agricultural management practices (Meriles et al., 2009; Liu et al., 2020). The primary agricultural practice of continuous cropping changes the soil microbial community structure (Zhang et al., 2018), decreasing bacterial biomass and increasing fungal biomass, leading to the dominant microorganisms altered from bacteria to fungi (Hu et al., 2010; Dong et al., 2016). Therefore, we need to adjust the soil properties used for continuous cropping by adding the necessary nutrients. Many strategies have been proposed to control continuous cropping problems, including crop rotation, soil sterilization and compost addition (Everts and Himmelstein, 2015; Xu et al., 2015; Ding et al., 2021). However, these strategies have their own limitations. Crop rotation takes a long time to play a regulatory role, soil sterilization might cause environmental damage, and compost releases nutrients slowly and has a low nutrient content (Grey and Henry, 1999; Bailey and Lazarovits, 2003; Tian et al., 2009). Microbial agents are composed of a large number of beneficial microorganisms, which can improve soil structure and affect plant growth (Boraste et al., 2009; Bhardwaj et al., 2014). The application of microbial agents can improve soil biological activity and the soil properties (Shah et al., 2001; Kolodziejczyk, 2014), so it is play a crucial role in sustainable agricultural development and are an indispensable technical measure for improving the quality of cultivated land (Ge et al., 2003). Supporters of microbial agent have been proving their beneficial effect on agricultural production for a long time (Xu, 2001). Therefore, forming bacteriostatic soil by increasing beneficial bacteria should suppress soil-borne diseases (Zhang et al., 2020). According to existing findings, we hypothesized that application of our microbial agent can promote the productivity of watermelon plants and improve soil quality, such as changing soil fertility, increasing beneficial microorganisms and decreasing harmful microorganisms.

Ding et al. recently reported that the application of urban waste compost alleviates soil problems associated with the continuous cropping of watermelon (Ding et al., 2021). However, there are few articles concerning the application of microbial agents for alleviating soil degradation caused by continuous cropping of watermelon. Thus, the purpose of this study was to assess the effectiveness of *Bacillus subtilis* and *Paecilomyces lilacinus,* these two strains exhibited inhibitory activity against a variety of plant pathogens (Li et al., 2022a,b), in alleviating soil degradation caused by continuous cropping and improving watermelon yield. We analyzed and compared soil chemical characteristics, microbial community structures, photosynthetic characteristics of watermelon leaves, and watermelon fruit quality and yield. Using high-throughput sequencing technology, we analyzed changes in soil microbial community structures to explore how microbial agents affect soil quality under continuous cropping.

## Materials and methods

### Source of microbial agent

The microbial agent contained two main components, *Paecilomyces lilacinus* DZ910 and *Bacillus subtilis* KC1723. *P. lilacinus* DZ910 was isolated from Zibo, Shandong Province, China. *B. subtilis* KC1723 was isolated from Jinan, Shandong Province, China. *P. lilacinus* DZ910 and *B. subtilis* KC1723 strains were deposited in the China General Microbiological Culture Collection Center under preservation numbers CGMCC 40079 and CGMCC 24397, respectively.

### Site descriptions and sampling

Watermelon was planted in a solar greenhouse located in Renfeng Town, Jinan City, China (37.16° N, 117.36° E) on January 13, 2022. The test site had a planting density of 36,000 plants per hm^2^ and has been used for growing watermelons for more than a decade. The soil is considered a fluvo-aquic type. The characteristics of the soil are as follows: pH, 7.76 ± 0.12; soil organic matter, 23.7 ± 0.6 g/kg. Ridge planting, conventional drip irrigation. All the treatments were managed in the same way.

The experiment consisted of four treatments: CK (untreated control), DZ910 (*P. lilacinus* DZ910), KC1723 (*B. subtilis* KC1723), D_K (mixture of *P. lilacinus* DZ910 and *B. subtilis* KC1723).Fungal strains were grown in PDA medium (*P. lilacinus* DZ910) and LB medium (*B. subtilis* KC1723). Conidial suspensions were prepared in sterile distilled water. Fungal conidial suspensions of *P. lilacinus* DZ910 (1 × 10^9^/mL), *B. subtilis* KC1723 (1 × 10^11^/mL), and a combined suspension of DZ910 and KC1723 (1 × 10^8^/mL + 1 × 10^10^/mL, respectively) were prepared and used for experiments. The fungal conidial suspension used in this study was 90 l/hm^2^, and diluted 10 times when used. Before transplanting, approximately 50 ml diluted suspension solution was poured into each hole. Watermelons of uniform size were used for this experiment. The daily field management of all treatments was consistent. Each treatment was replicated three times.

### Photosynthetic efficiency and virulence assays

Three months after application of different microbial agents, chlorophyll content and photosynthesis parameters were measured using a portable instrument (Handy PEA, Hansatech). Three partitioned blades were tested, the lower blade (LB) was 50 cm away from the ground, the middle blade (MB) was 100 cm away, and the upper blade (UB) was 150 cm away. The experiment was repeated three times independently with 20 replicates each.

Virulence assays were performed at the same time. Ten leaves per plant were examined from 50 cm above the ground. According to the proportion of diseased area to total area, the disease index (DI) was classified into the following grades: 1, ≤5%; 3, 6–10%; 5, 11–25%; 7, 26–50%; 9, ≥50%. DI = Σ(number of disease plants×corresponding disease grade value)/(total number of plants investigated×highest value) × 100. The experiment was repeated three times independently with 15 replicates each.

### Yield and fruit quality

Ten plants from each treatment were randomly assigned to assess watermelon fruit on April 19, 2022. The fruit were weighed and then used for quality determination. Fruit firmness, soluble solid content and sugar content were detected using the appropriate instruments following the corresponding operation instructions.

### Determination of soil physicochemical properties

Total nitrogen (TN) contents were measured using an automatic Kjeldahl distillation titration unit (Foss, Sweden). Total phosphorus (TP) was digested with H_2_SO_4_-HClO_4_ (Miranda et al., 2001). Total potassium (TK) was digested with HNO_3_-HClO_4_-HF (Shen et al., 2013). Soil total organic matter (OM) was assayed according to the vitriol acid potassium dichromate oxidation method (Nelson and Sommers, 2018).

### DNA extraction and sequencing of soil microbial communities

Soil total DNA was extracted from 500 mg of fresh soil using a PowerSoil® DNA Isolation Kit (MO Bio Laboratories, San Diego, CA, USA) according to the manufacturer’s protocol. The bacterial universal primer pair 338F (5′-GTACTCCTACGGGA GGCAGCA-3′) and 806R (5′-GTGGACTACHVGGGTW TCTAAT-3′) targeting the V3-V4 region of the 16S rRNA gene was used to characterize soil bacterial communities (Kim et al., 2011). The fungal universal primer pair ITS1-F (5′-CTTGGTCATTTAGAGGAAGTAA-3′) and ITS2-R (5′-TGCGTTCTTCATCGATGC-3′) targeting the internal transcribed spacer (ITS) region was used to identify soil fungal communities (Martin, 2011).

The 16S rRNA gene and ITS regions in the DNA samples were amplified by polymerase chain reaction (PCR) before sequencing. The PCR was performed in a total volume of 50 μl, containing 2 μl of template DNA, 5 μl of 10 × buffer, 2 μl of 10 μM forward primer, 2 μl of 10 μM reverse primer, 4 μl of 2.5 μM dNTPs, 0.3 μl of DNA polymerase (2.5 U μL^−1^), and 34.7 μl of ddH2O. Reaction conditions were 5 min at 95°C, followed by 30 cycles (for the bacterial 16S rRNA gene) or 35 cycles (for the fungal ITS region) of 30 s at 95°C, 30 s at 56°C and 40 s for elongation at 72°C, followed by a final extension at 72°C for 10 min and a hold at 10°C.

All PCR amplicons were examined using 2% (w/v) agarose gels. Purified DNA was quantified using a QuantiFluor™-ST (Promega, USA) and subsequently sequenced (PE300) using the Illumina MiSeq platform (Illumina Inc., USA) by Shanghai Majorbio Bio-pharm Technology Co., Ltd. Operational taxonomic units (OTUs) were assigned at a threshold of 97% similarity level and clustered using UPARSE (version 7.1) (Edgar, 2013). Chimeric sequences were identified and removed using UCHIME (Knight, 2011). Representative sequences of each OTU were taxonomically classified using the Silva and Unite database (Quast et al., 2012). Sequences matching the archaea, mitochondria and chloroplasts were removed and the number of sequences were rarefied to 32,650 reads per sample. The sequencing datasets supporting the current study are available in the National Center for Biotechnology Information (NCBI) under BioProject accession number PRJNA908896.

### Data analysis

Data were analyzed by one-way analysis of variance (ANOVA) using SPSS 20.0 (SPSS Inc., USA). Alpha-diversity analyses, including ACE index, Chao index and Shannon index, were calculated using Mothur software (Quast et al., 2012). Taxonomic dissimilarity analysis between samples was based on the PCoA method with Bray-Curtis distances (beta diversity) (Lozupone et al., 2011). The R language vegan package (version 3.3.1) was used to draw community heat maps. The relationship between genus diversity and soil physicochemical properties was analyzed using redundancy analysis (RDA) by Canoco for Windows (ver. 4.5). The significance level for differences was set at *p* ≤ 0.05.

## Results

### Effects of microbial agents on watermelon agronomic variables

**Figure 1:**
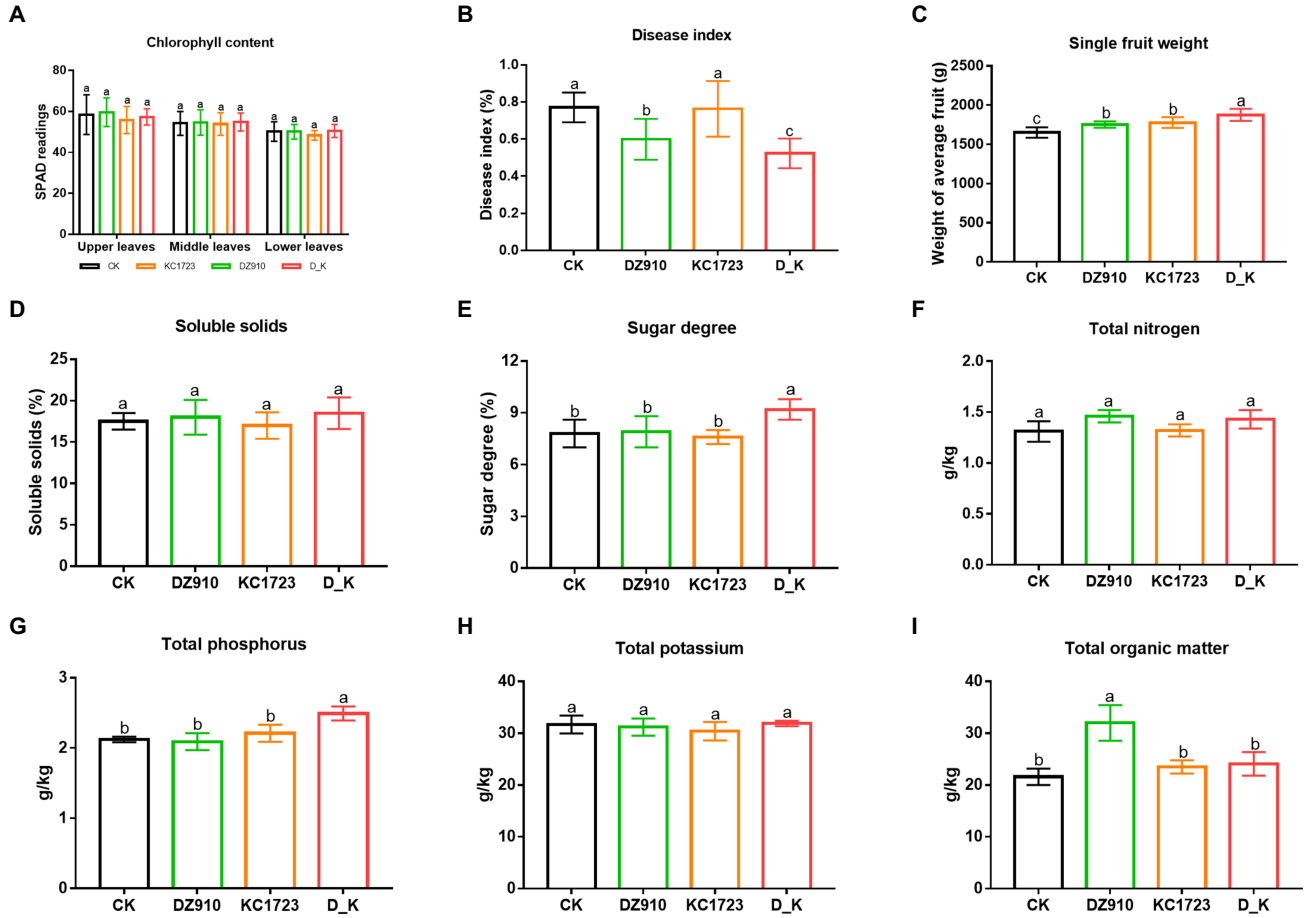
Changes of watermelon performance and soil physicochemical properties in different treatments. **(A)** chlorophyll content, **(B)** disease index, **(C)** SWF, **(D)** soluble solids, **(E)** sugar degree, **(F)** total nitrogen, **(G)** total phosphorus, **(H)** total potassium, and **(I)** total organic matter. The letters above the values indicate significant differences in different treatments (analysis of variance [ANOVA]: Duncan test; *p* < 0.05).

We measured chlorophyll content, photosynthetic efficiency and disease index of leaves to assess the growth of plants under different treatment (Figures 1A,B; Supplementary Figure S1a). Microbial agents had no obvious effect on chlorophyll content (Figures 1A). The photosynthetic efficiency of upper leaves (PE-up) and lower leaves (PE-low) were significantly higher under *P. lilacinus* (DZ910), *B. subtilis* (KC1723) and mixed microbial agent (D_K) treatments than in the control (CK) (*p* < 0.05) (Supplementary Figure S1a). The disease index of leaves was significantly lower under DZ910 and D_K treatments than under CK and KC1723 treatments (p < 0.05) (Figure 1B).

The single fruit weight (SWF), soluble solids and sugar degree were determined to assess the growth of watermelon fruit (Figures 1C–E; Supplementary Figure S1b). The D_K treatment produced the highest SWF, which was significantly higher than that in other treatments (Figure 1C; Supplementary Figure S1b). Sugar degree was significantly higher in the D_K treatment compared with the other treatments (*p* < 0.05) (Figure 1E). However, there were no significant differences in the soluble solids of fruit among different treatments (Figure 1D).

### Changes in soil physicochemical properties

The effects of using *P. lilacinus* (DZ910), *B. subtilis* (KC1723) and the mixed microbial agent (D_K) on soil physicochemical characteristics are shown in Figure 1. Contents of TN and TK did not differ significantly between treatments (Figures 1F,G). TP in the D_K treatment was significantly higher than that in the other treatments (Figure 1H). Likewise, soil OM in the DZ910 treatment was significantly higher than that in other treatments (Figure 1I).

### Soil bacterial and fungal alpha diversities

**Figure 2:**
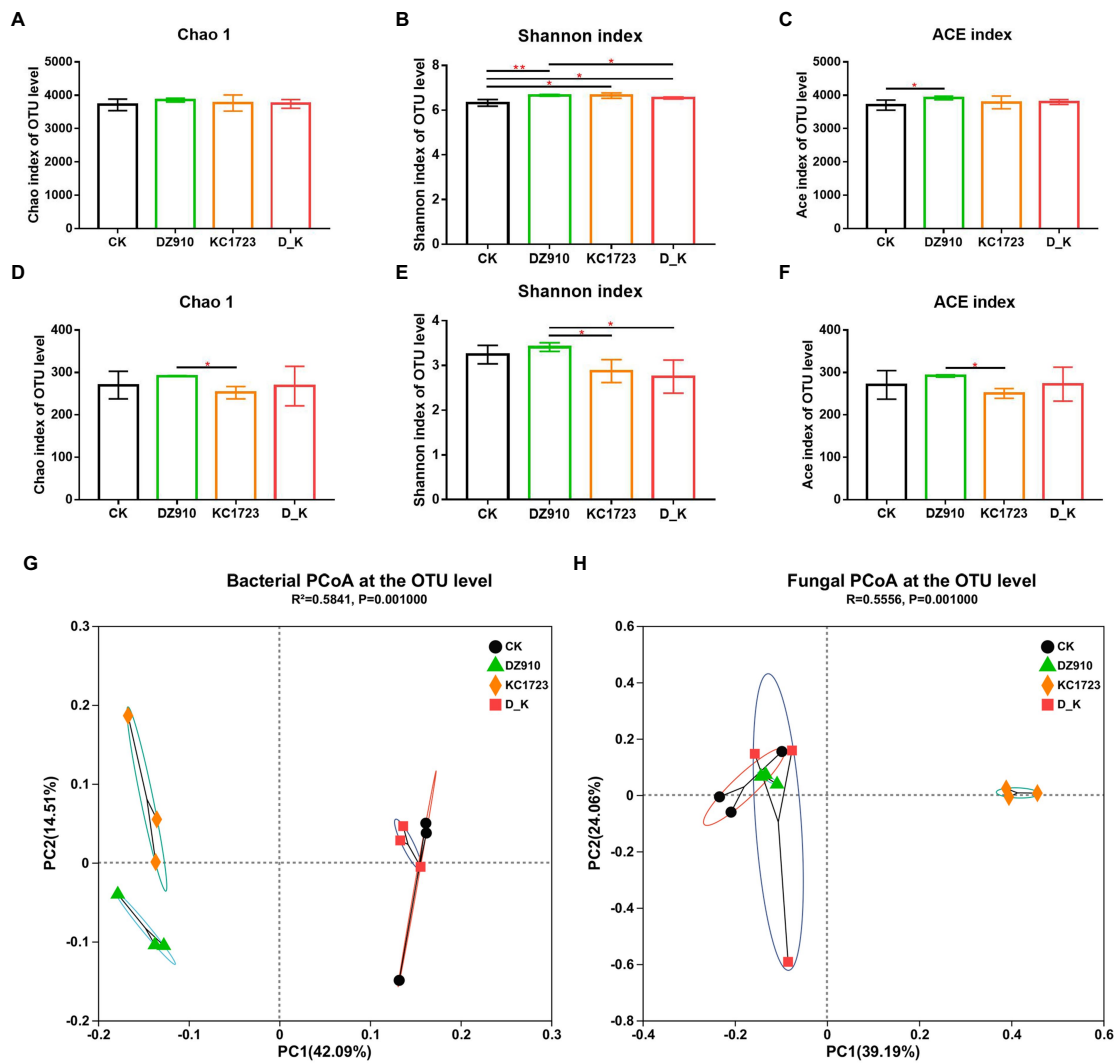
Changes in soil microbial alpha and beta diversities. **(A–C)** bacterial Chao1, Shannon and ACE indices, **(D–F)** fungal Chao1, Shannon and ACE indices, **(G)** PCoA clustering analysis of bacteria, **(H)** PCoA clustering analysis of fungi. Error bars indicate mean ± SE. *p*-values by *T*-test indicated significant differences, and the symbol (*) indicated that 0.01 < *p* ≤ 0.05, (**) indicated that 0.001 < *p* ≤ 0.01.

We evaluated and compared the richness and diversity of bacterial and fungal communities in the different soil samples using Chao1, Shannon and ACE indices (Figure 2). As shown in Figures 2A,D, the Chao1 estimator for the bacterial community showed no significant difference between samples, whereas a lower Chao1 estimator for the fungal community was observed in the soil treated with KC1723. The Shannon index of the bacterial community was higher in the sample under DZ910, KC1723 and D_K treatments than under CK, with the highest value in the soil treated with DZ910 (Figure 2B). However, the Shannon index for the fungal community showed a lower value in the soil under KC1723 and D_K treatment (Figure 2E). The ACE index value for the bacterial community was significantly higher under DZ910 treatment than under other treatments, while the ACE index value for the fungal community was significantly lower under KC1723 treatment than under other treatments (Figures 2C,F).

### Soil bacterial and fungal beta diversities

The results of principal coordinate analysis (PCoA) of different soil samples are also shown in Figure 2. Variation in bacterial and fungal community composition was affected by 11 principal coordinate components, respectively (Figures 2G,H). In terms of bacteria, the cumulative contribution rates of the two maximally reflected differences were 42.09 and 14.51% and the cumulative explanatory variation was 56.6% (Figure 2G). The distance between CK and D_K treatments was small, and they grouped together. DZ910 and KC1723 treatments were both far away from CK and D_K treatments. In fungi, the two main coordinates extracted explained 63.25% of the variation, of which 39.19% was explained by PC1 and 24.06% by PC2 (Figure 2H). Similarly, the results showed that CK, DZ910 and D_K treatments gathered together. However, the KC1723 treatment was far away from CK, DZ910 and D_K treatments.

### Microbial community structures of different treatments

**Figure 3:**
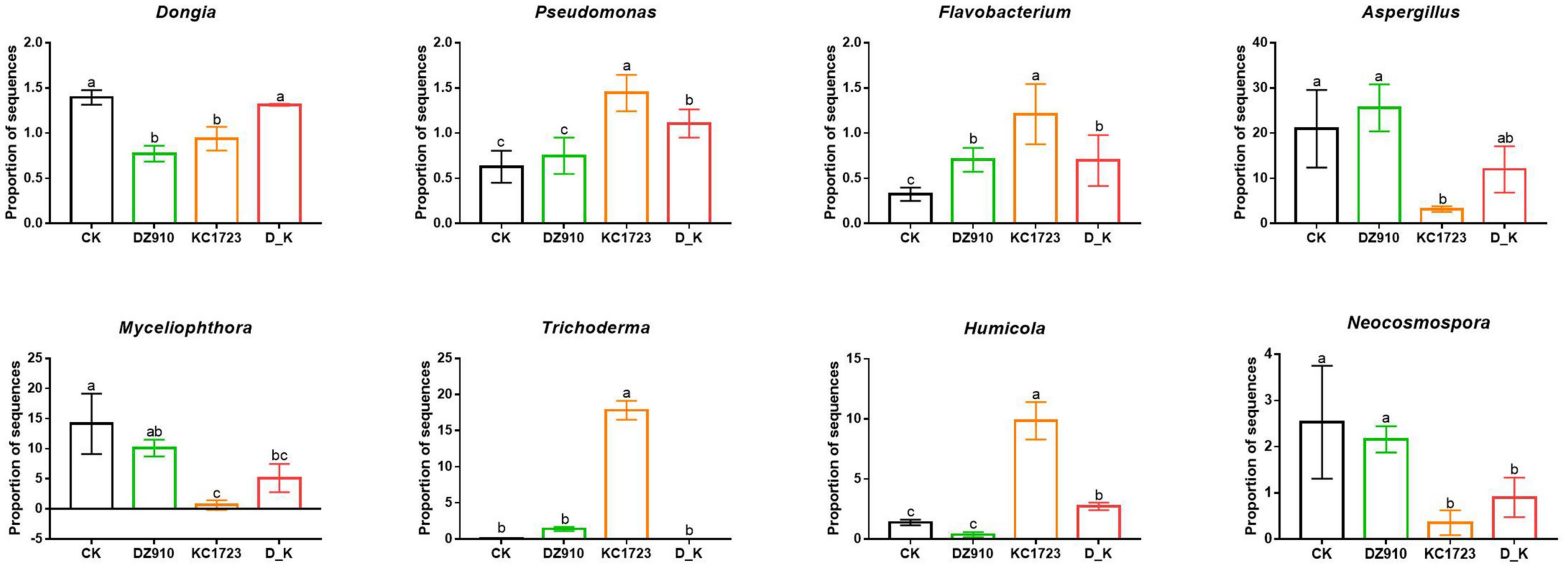
Effects of different microbial agents on several key microorganisms. The letters indicate significant differences among the different treatments (analysis of variance [ANOVA]: Duncan test; *p* < 0.05).

The bacterial and fungal populations in the different treatments were analyzed by high-throughput sequencing. In this study, a total of 26 bacterial genera and 20 fungal genera (relative abundance >1%) were detected in the different soil samples (Supplementary Tables S1, S2). To further analyze the relative abundance of the microbial community, we found that 3 identified bacterial genera, including *Dongia*, *Pseudomonas* and *Flavobacterium*, and five identified fungal genera, *Aspergillus*, *Myceliophthora*, *Trichoderma*, *Humicola* and *Neocosmospora*, showed significant differences in the soil under different treatments (Supplementary Tables S1, S2; Figure 3). In the bacterial community, OTUs belonging to *Dongia* were decreased in DZ910 and KC1723 treatments, but showed no significant change in D_K compared with those in CK (Figure 3). The abundance of *Pseudomonas* increased significantly in KC1723 and D_K, and the abundance of *Flavobacterium* increased significantly in all microbial agent treatments (DZ910, KC1723 and D_K) compared with those in CK (Figure 3). Within the fungal community, the abundance of *Aspergillus* decreased significantly in KC1723 and the abundance of *Myceliophthora* and *Neocosmospora* decreased significantly in KC1723 and D_K compared to those in CK (Figure 3). However, the abundance of *Trichoderma* and *Humicola* increased significantly in the KC1723 treatment compared to CK (Figure 3).

### Relationships of microbial community with physicochemical properties

**Figure 4:**
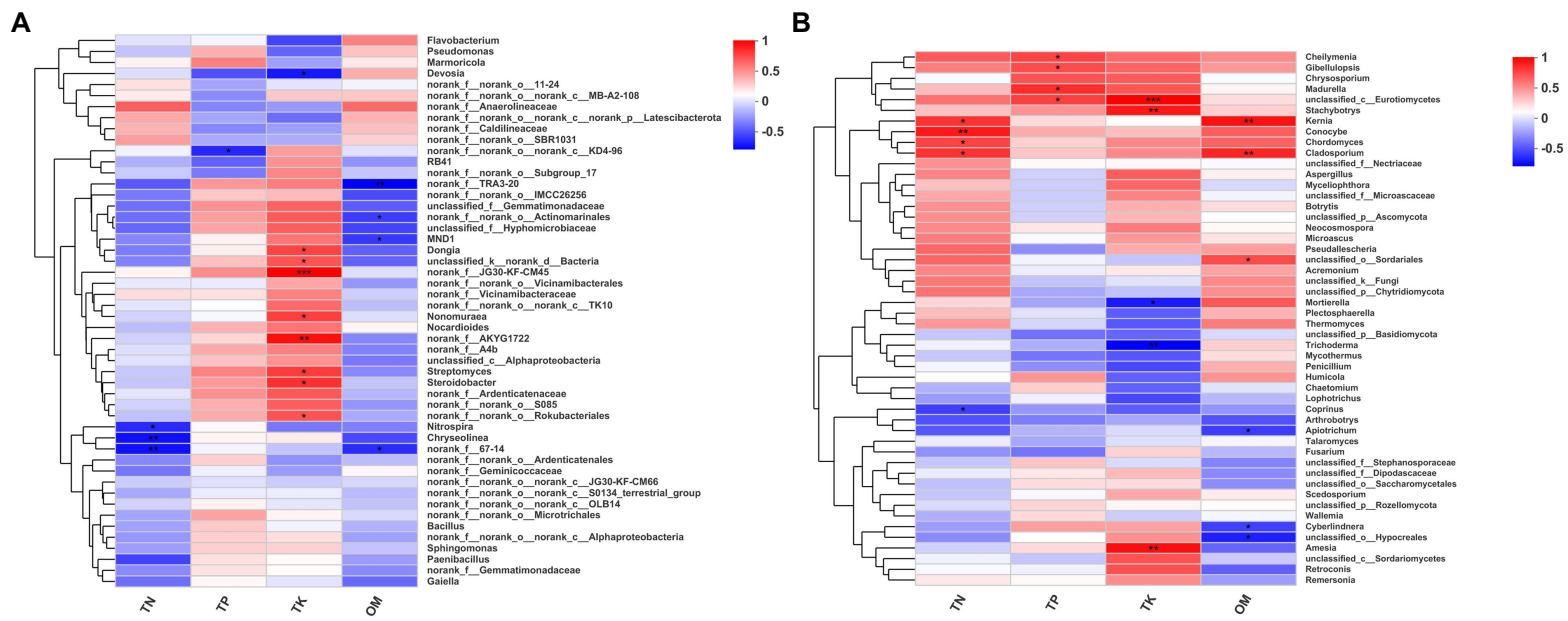
Correlation heat map of microbial classification and environmental factors. **(A)** Correlation heat map of the top fifty genera (bacteria) and environmental factors. **(B)** Correlation heat map of the top fifty genera (fungi) and environmental factors. The symbol (*) indicated that 0.01 < *p* ≤ 0.05, (**) indicated that 0.001 < *p* ≤ 0.01, (***) indicated that *p* ≤ 0.001.

We constructed a correlation heatmap between microbial community at the genus level and physicochemical properties using Spearman’s correlation analysis (Figure 4). Among the top 50 most abundant bacterial genera, 16 genera were affected by at least one physicochemical property (Figure 4A). *Nitrospira*, *Chryseolinea* and *norank_f_67–14* were significantly negatively correlated with TN. *Norank_f_norank_o_norank_c_KD4-96* was significantly negatively correlated with TP. Nine genera were affected by TK, one of which was negatively affected by TK, while the other eight genera, *Dongia*, *unclassified_k_norank_d_Bacteria*, *norank_f_JG30-KF-CM45*, *Nonomuraea*, *norank_f_AKYG1722*, *Streptomyces*, *Steroidobacter* and *norank_f_norank_o_Rokubacteriales*, were positively correlated with TK. Four genera, *norank_f_TRA3-20*, *norank_f_norank_o_Actinomarinales*, *MND1* and *norank_f_67–14*, were significantly negatively correlated with OM.

In the top 50 fungal genera, 17 genera were affected by at least one physicochemical property (Figure 4B). *Kernia*, *Conocybe*, *Chordomyces* and *Cladosporium* were significantly positively correlated with TN, and *Coprinus* was significantly negatively correlated with TN. *Cheilymenia*, *Gibellulopsis*, *Madurella* and *unclassified_c_Eurotiomycetes* were significantly positively correlated with TP. Three genera, *unclassified_c_Eurotiomycetes*, *Stachybotrys* and *Amesia*, were significantly positively affected by TK, and two genera, *Mortierella* and *Trichoderma* were significantly negatively affected by TK. Six genera were affected by OM: *Kernia*, *Cladosporium*, *unclassified_o_Sordariales*, *Apiotrichum*, *Cyberlindnera* and *unclassified_o_Hypocreales*.

### Network analysis of physicochemical properties, major microorganisms and agronomic variables

**Figure 5:**
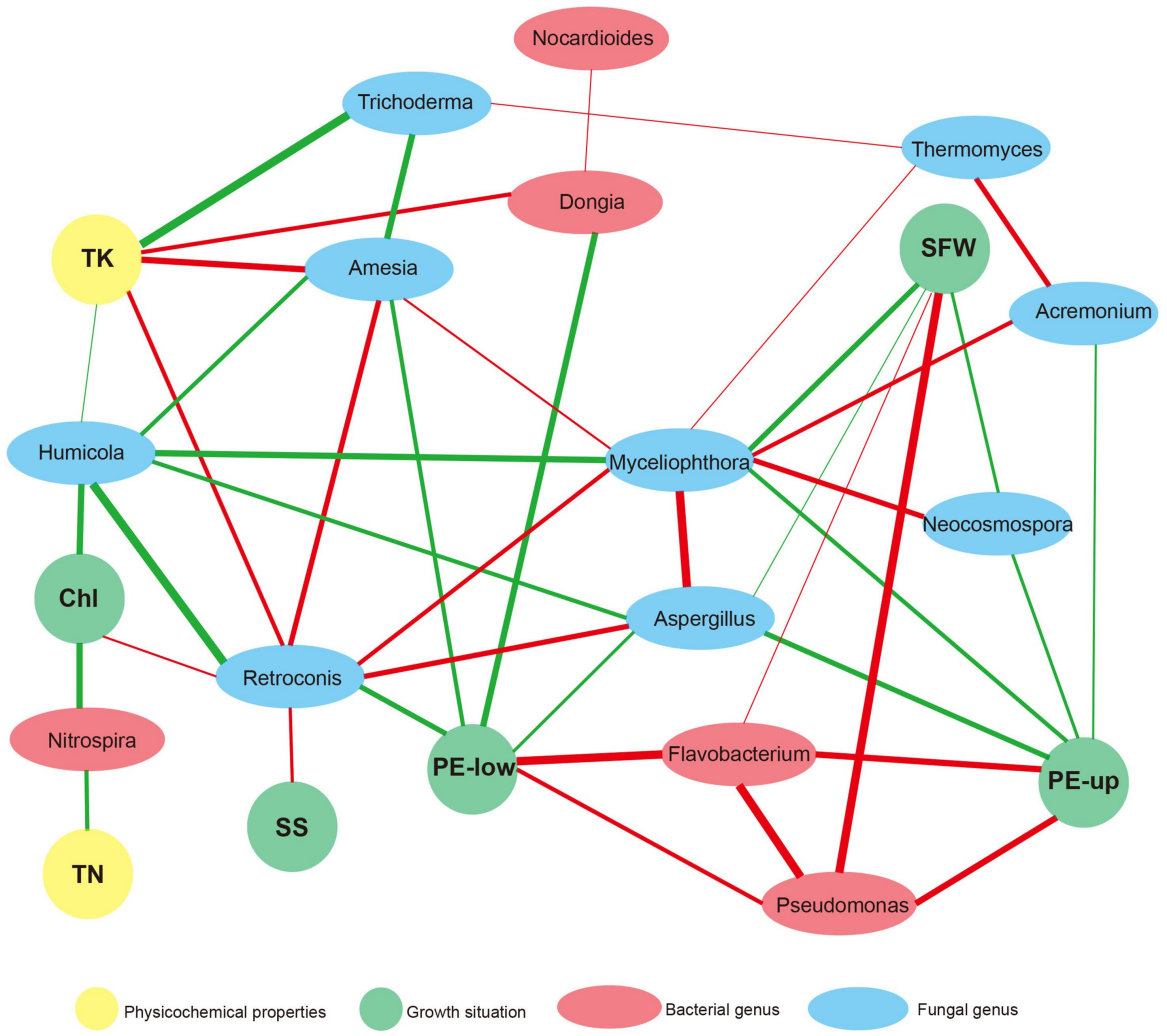
Network analysis based on the co-occurrence of physicochemical properties, major microorganisms and the agronomic variables. The agronomic variables, such as PE-up and PE-low leaves, SFW, sugar degree (SS) and chlorophyll content (Chl), were shown in green ellipses. The physicochemical properties, TK and TN were shown in yellow ellipses. The major bacteria, including *Dongia*, *Pseudomonas*, *Flavobacterium*, *Nitrospira*, and *Nocardioides*, were shown in red ellipses, and the major fungus, such as *Aspergillus*, *Myceliophthora*, *Trichoderma*, *Humicola*, *Neocosmospora*, *Retroconis*, *Acremonium*, *Amesia* and *Thermomyces*, were shown in gray ellipses. A connection represents a significant correlation (*p* < 0.05) according to Spearman’s analysis. The red lines denote positive correlations and the green lines mean negative correlations (the darker the color the stronger the correlation).

We conducted network analysis among physicochemical properties, major microorganisms and agronomic variables to assess their relationships. There were 22 nodes and 44 edges in the network (Figure 5). TK was the physicochemical characteristic most related to microorganisms. It was positively related to *Dongia*, *Amesia* and *Retroconis,* and negatively related to *Trichoderma* and *Humicola*. TN was strongly associated with *Nitrospira*, which was similar to the results of the correlation heatmap (Figure 5). Interestingly, two bacterial genera, *Pseudomonas* and *Flavobacterium*, were positively related to SWF, PE-up and PE-low. At the same time, a positive correlation was observed between them. The fungal genus *Myceliophthora* was positively related to *Neocosmospora* and *Aspergillus*, and they were all negatively related to SWF and PE-up.

## Discussion

Previous studies indicated that obstacles to continuous cropping are caused by long-term monoculture cropping leading to a reduction in soil quality and crop productivity (Qin et al., 2017; Chen et al., 2020). There are many complex factors contributing to obstacles to continuous cropping, including changes in soil physicochemical properties, increases in autotoxic substances and changes in microbial communities in soil (Li et al., 2018; Li and Liu, 2019). Application of microbial agents is one way to relieve soil sickness and increase the yield and quality of agricultural products (Mandrik et al., 2020). In this study, application of our microbial agents effectively improved the growth of watermelon and soil degradation caused by continuous cropping.

Chlorophyll content, photosynthetic efficiency and disease index help to assess the growth of crops (Cheng et al., 2012). In this study, three types of microbial agents increased the PE-up and PE-low, which helped to improve the growth of watermelon. The microbial agents are reported to promote photosynthesis, thereby increasing net photosynthetic rate (Agamy et al., 2012; Duan et al., 2021). As shown in Figures 1C,E, both SWF and sugar degree were higher under D_K treatment than under other treatments. This indicates that *B. subtilis* and *P. lilacinus* synergistically promote plant photosynthetic efficiency, resulting in increased SWF and sugar degree.

Soil physicochemical properties (TN, TP, TK and OM) are very important properties in soil ecosystems that not only reflect soil health and quality, but also act as ideal indicators for assessing the growth of crops (Lemaire et al., 2019; Chen et al., 2020). TP improved significantly after applying a mixture of *P. lilacinus* DZ910 and *B. subtilis* KC1723 (D_K) (Figures 1F–I). K fertilizer is a essential nutrient in soil for plant growth and plays vital roles in the yield and quality of agricultural crops (Song et al., 2020). In this study, application of D_K might have increased soil total phosphorous and promoted watermelon growth.

Microorganisms also play a vital role in maintaining plant health (Cardinale et al., 2006; Diacono and Montemurro, 2011). Changes in the diversity and composition of soil microorganisms has a considerable influence on soil ecosystems (Alami et al., 2021). Application of different microbial agents had a significant influence on the composition, diversity and structure of the bacterial and fungal communities in the soil (Figures 2, 3; Supplementary Tables S1, S2). Only three bacterial genera (greater than 1%), *Dongia*, *Pseudomonas* and *Flavobacterium*, showed significant differences under different treatments (Figure 3). Network analysis showed that *Pseudomonas* and *Flavobacterium* were positively related to the growth of watermelon (Figure 5). Application of KC1723 and D_K significantly increased the relative abundance of *Pseudomonas*. Previous studies found that *Pseudomonas* is a plant-beneficial bacteria, and its abundance is significantly depleted after continuous cropping (Rumberger, 2007; Xiong et al., 2015). At the same time, the application of microbial agents also significantly increased the relative abundance of *Flavobacterium* in soil. This is thought to be correlated with the capacity to degrade complex organic compounds (Kolton et al., 2016). The abundance of *Flavobacterium* in rhizosphere soil is positively related to fruit maturation and plant resistance (Sang and Kim, 2012; Kolton et al., 2014; Zhou et al., 2022). Therefore, *Pseudomonas* and *Flavobacterium* might be significant factors for watermelon growth. Five fungal genera (greater than 1%), *Aspergillus*, *Myceliophthora*, *Trichoderma*, *Humicola* and *Neocosmospora*, showed significant differences after the application of different microbial agents (Figure 3). Network analysis indicated that *Aspergillus*, *Myceliophthora* and *Neocosmospora* are negatively related to SWF and PE-up (Figure 5). Chen et al. found that the ratio of *Aspergillus* increased in soil of *Panax quinquefolium* following continuous cropping (Chen et al., 2012). In our study, application of KC1723 significantly decreased the abundance of *Aspergillus* in the soil. The mix microbial agent D_K contains KC1723, and could also reduce the content of *Aspergillus* in the soil. *Neocosmospora* is an important genus of phytopathogens, causing stem rot in many crops, such as soybean and potato (Greer et al., 2015; Riaz et al., 2022a,b). This fungus symptomatically causes stunted growth and typical grayish-black streaking on plants (Riaz et al., 2022a,b). With the spread of *Neocosmospora* fungus, plants gradually wilt and then die (Riaz et al., 2020). In our study, application of KC1723 and D_K effectively reduced the abundance of *Neocosmospora* in watermelon continuous cropping soil, which probably explains why using microbial agents, especially KC1723 and D_K, can alleviate problems with watermelon continuous cropping.

Collectively, microbial agents have great potential in continuous cropping soil remediation. In this study, microbial agents (DZ910, KC1723 and D_K) improved watermelon growth, soil physicochemical properties and microbial community structures. The D_K treatment, which combined suspensions of DZ910 and KC1723, showed the best continuous cropping soil remediation effect.

## Conclusion and perspectives

This study revealed that application of microbial agents (DZ910, KC1723 and D_K) can relieve the obstacles of continuous cropping to watermelon cultivation. Agronomic variables of watermelon, such as PE-up and PE-low, SWF and sugar degree of fruit, and TP of soil under D_K treatment changed significantly. Soil microbial communities under microbial agent treatments also changed significantly, indicating the feasibility of microbial agents as soil remediations. Levels of several bacteria in the soil, such as *Pseudomonas* and *Flavobacterium*, and fungi, *Aspergillus* and *Neocosmospora*, changed significantly after using microbial agents. Besides, *Pseudomonas* and *Flavobacterium* were positively correlated with the growth of watermelon. *Aspergillus* and *Neocosmospora* were positively related to *Myceliophthora* and negatively correlated with watermelon growth (SWF and PE-up). Above all, our results provide a useful technique for relieving obstacles to continuous cropping of watermelon.

## Data availability statement

The datasets presented in this study can be found in online repositories. The names of the repository/repositories and accession number(s) can be found below: NCBI Sequence Read Archive Project - PRJNA908896.

## Author contributions

JL and XG conceived and designed the research. PC, JZ, ML, JH, AZ, ZS, and FF performed the experiments. PC, JH, ZS, and AZ collected samples and performed data acquisition. PC, JZ, and JL analyzed the data and wrote the main manuscript text. All authors contributed to the article and approved the submitted version.

## Funding

This research was supported by the National Key Research and Development Program of China (no. 2021YFD1700101) and Key Technology Research and Development Program of Shandong (no. 2021CXGC010811).

## Conflict of interest

The authors declare that the research was conducted in the absence of any commercial or financial relationships that could be construed as a potential conflict of interest.

## Publisher’s note

All claims expressed in this article are solely those of the authors and do not necessarily represent those of their affiliated organizations, or those of the publisher, the editors and the reviewers. Any product that may be evaluated in this article, or claim that may be made by its manufacturer, is not guaranteed or endorsed by the publisher.
