# Supplementary figures and images for: Synergistic effect of Bacillus subtilis and Paecilomyces lilacinus in alleviating soil degradation and improving watermelon yield
Source: Front Microbiol. 2023 Jan 13;13:1101975. doi: 10.3389/fmicb.2022.1101975 (PMC9881412; doi:10.3389/fmicb.2022.1101975)

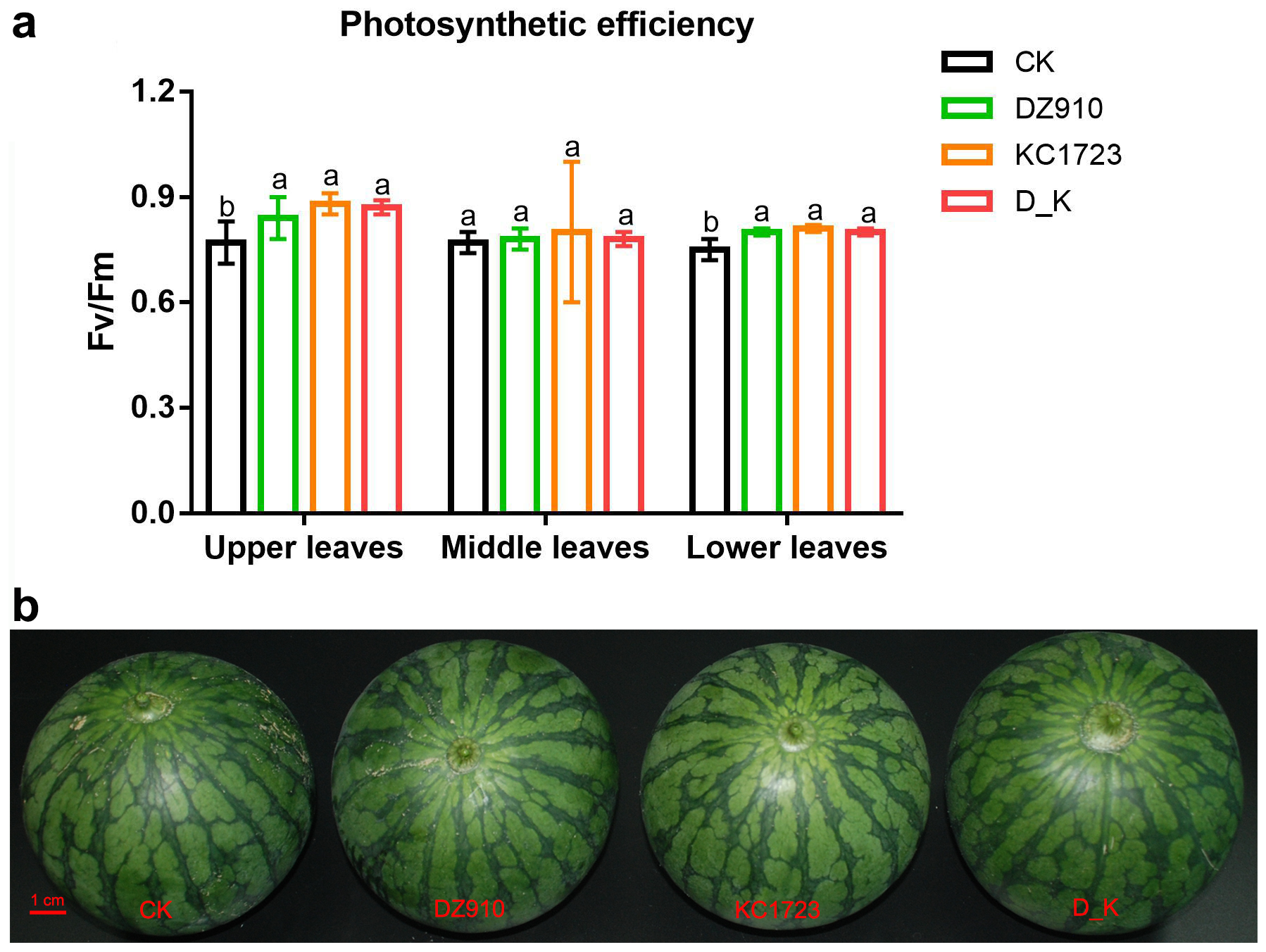

Supplement: Supplementary file 1 [file Image_1.TIF]
