# Supplementary material for: Synergistic effect of Bacillus subtilis and Paecilomyces lilacinus in alleviating soil degradation and improving watermelon yield
Source: Front Microbiol. 2023 Jan 13;13:1101975. doi: 10.3389/fmicb.2022.1101975 (PMC9881412; doi:10.3389/fmicb.2022.1101975)
Supplement: Supplementary file 2 [file Table_1.docx]

Table S1 Composition of the different bacterial communities at the genus level in different treatments(relative abundance > 1%).The symbol (*) indicated that 0.01<P≤0.05, (**) indicated that 0.001<P≤0.01, (***) indicated that P≤0.001.

| Genus name | CK | DZ910 | KC1723 | D_K | Pvalue |
| --- | --- | --- | --- | --- | --- |
|  | Mean±Sd (%) | Mean±Sd (%) | Mean±Sd (%) | Mean±Sd (%) |  |
| Bacillus | 7.49±1.46 | 7.20±0.73 | 8.82±2.34 | 7.50±0.36 | 0.790 |
| norank_f__norank_o__Vicinamibacterales | 5.42±2.37 | 4.84±1.51 | 2.78±1.51 | 3.97±0.07 | 0.507 |
| RB41 | 4.39±0.40 | 4.07±1.35 | 2.61±1.53 | 3.18±0.53 | 0.150 |
| norank_f__Vicinamibacteraceae | 4.51±2.69 | 4.06±0.94 | 2.22±1.12 | 3.42±0.03 | 0.408 |
| norank_f__A4b | 3.56±0.62 | 3.25±0.45 | 2.97±0.65 | 3.75±0.35 | 0.429 |
| norank_f__Gemmatimonadaceae | 2.33±0.47 | 2.29±0.04 | 2.59±0.17 | 2.48±0.27 | 0.209 |
| Streptomyces | 2.19±0.86 | 1.15±0.03 | 1.51±0.47 | 2.51±0.74 | 0.134 |
| norank_f__norank_o__norank_c__Alphaproteobacteria | 1.43±0.40 | 1.20±0.13 | 1.59±0.18 | 1.39±0.06 | 0.193 |
| norank_f__norank_o__Actinomarinales | 1.39±0.10 | 0.94±0.10 | 1.11±0.06 | 1.36±0.08 | 0.014* |
| norank_f__norank_o__S085 | 1.18±0.07 | 1.17±0.13 | 1.04±0.15 | 1.38±0.13 | 0.201 |
| norank_f__Caldilineaceae | 0.89±0.08 | 1.66±0.29 | 1.08±0.09 | 0.90±0.04 | 0.041* |
| Dongia | 1.40±0.08 | 0.77±0.09 | 0.94±0.13 | 1.31±0.01 | 0.005** |
| norank_f__JG30-KF-CM45 | 1.15±0.16 | 0.89±0.05 | 0.85±0.13 | 1.47±0.26 | 0.079 |
| norank_f__norank_o__norank_c__TK10 | 1.02±0.14 | 1.05±0.04 | 0.86±0.09 | 1.11±0.04 | 0.072 |
| norank_f__Ardenticatenaceae | 0.97±0.08 | 0.91±0.12 | 0.85±0.17 | 1.21±0.12 | 0.113 |
| Pseudomonas | 0.63±0.18 | 0.75±0.20 | 1.45±0.20 | 1.11±0.16 | 0.026* |
| MND1 | 1.18±0.32 | 0.91±0.10 | 0.87±0.08 | 0.96±0.04 | 0.458 |
| norank_f__norank_o__Ardenticatenales | 0.77±0.10 | 0.91±0.15 | 1.14±0.03 | 0.96±0.04 | 0.008* |
| norank_f__norank_o__norank_c__S0134_terrestrial_group | 0.85±0.14 | 0.90±0.08 | 1.01±0.16 | 0.88±0.13 | 0.727 |
| Gaiella | 0.90±0.21 | 0.77±0.11 | 1.00±0.11 | 0.85±0.27 | 0.283 |
| norank_f__norank_o__Rokubacteriales | 1.01±0.33 | 0.76±0.11 | 0.75±0.25 | 1.00±0.03 | 0.127 |
| norank_f__norank_o__norank_c__KD4-96 | 0.99±0.03 | 1.04±0.25 | 0.64±0.36 | 0.79±0.06 | 0.046* |
| norank_f__AKYG1722 | 1.06±0.20 | 0.74±0.02 | 0.63±0.05 | 0.95±0.16 | 0.054 |
| norank_f__Geminicoccaceae | 0.71±0.14 | 0.66±0.05 | 1.14±0.19 | 0.79±0.14 | 0.088 |
| norank_f__Anaerolineaceae | 0.23±0.14 | 1.60±0.43 | 0.78±0.44 | 0.32±0.05 | 0.048* |
| Flavobacterium | 0.32±0.07 | 0.71±0.13 | 1.21±0.33 | 0.70±0.28 | 0.028* |
