# Supplementary material for: Synergistic effect of Bacillus subtilis and Paecilomyces lilacinus in alleviating soil degradation and improving watermelon yield
Source: Front Microbiol. 2023 Jan 13;13:1101975. doi: 10.3389/fmicb.2022.1101975 (PMC9881412; doi:10.3389/fmicb.2022.1101975)
Supplement: Supplementary file 3 [file Table_2.docx]

Table S2 Composition of the different fungal communities at the genus level in different treatments (relative abundance > 1%).The symbol (*) indicated that 0.01<P≤0.05, (**) indicated that 0.001<P≤0.01, (***) indicated that P≤0.001.

| Genus name | CK | DZ910 | KC1723 | D_K | Pvalue |
| --- | --- | --- | --- | --- | --- |
|  | Mean±Sd (%) | Mean±Sd (%) | Mean±Sd (%) | Mean±Sd (%) |  |
| Mortierella | 15.25±4.20 | 25.72±3.30 | 33.92±8.34 | 12.73±6.40 | 0.052 |
| Aspergillus | 20.98±8.61 | 25.59±5.19 | 3.17±0.63 | 11.98±5.15 | 0.014* |
| Myceliophthora | 14.13±5.01 | 10.10±1.39 | 0.65±0.80 | 5.12±2.33 | 0.003** |
| Cheilymenia | 7.04±12.2 | 0.004±0.003 | 0.00±0.00 | 17.86±15.47 | NA |
| Chrysosporium | 7.95±3.88 | 2.59±0.34 | 3.73±0.80 | 8.01±4.61 | 0.139 |
| unclassified_f__Microascaceae | 8.50±4.89 | 7.04±0.53 | 3.11±0.48 | 3.44±0.77 | 0.004** |
| Chaetomium | 4.05±0.35 | 3.36±0.29 | 11.62±5.99 | 2.51±0.96 | 0.103 |
| Trichoderma | 0.04±0.02 | 2.12±1.39 | 17.82±1.32 | 0.02±0.01 | 0.0001*** |
| Humicola | 1.39±2.34 | 0.37±0.21 | 9.85±1.56 | 2.73±3.16 | 0.008** |
| unclassified_f__Dipodascaceae | 1.45±1.84 | 1.44±1.83 | 0.12±0.08 | 10.56±17.91 | 0.498 |
| unclassified_o__Saccharomycetales | 0.81±1.40 | 0.03±0.03 | 0.002±0.002 | 10.31±17.83 | 0.461 |
| unclassified_k__Fungi | 2.04±0.57 | 5.04±2.45 | 1.74±0.97 | 1.10±0.61 | 0.199 |
| unclassified_f__Nectriaceae | 1.78±0.47 | 2.20±0.41 | 4.88±7.35 | 0.82±0.19 | 0.034* |
| Neocosmospora | 2.53±1.22 | 2.16±0.29 | 0.36±0.27 | 0.90±0.43 | 0.008** |
| Arthrobotrys | 1.93±1.47 | 1.20±0.25 | 1.63±0.86 | 0.96±1.03 | 0.771 |
| unclassified_o__Sordariales | 0.50±0.12 | 1.46±0.08 | 0.58±0.27 | 0.76±0.56 | 0.002** |
| Plectosphaerella | 0.61±0.34 | 1.24±0.61 | 0.97±0.47 | 0.32±0.18 | 0.180 |
| Pseudallescheria | 0.80±0.42 | 1.14±0.26 | 0.41±0.12 | 0.54±0.14 | 0.067 |
| Cyberlindnera | 0.24±0.40 | 0.003±0.006 | 0±0 | 2.11±3.63 | NA |
| Madurella | 0.18±0.30 | 0.005±0.009 | 0±0 | 1.76±3.02 | NA |
